# High-Risk Analysis of Vertebral Compression Fractures With Type 2 Diabetes Mellitus: Site-Specific Volumetric Bone Mineral Density

**DOI:** 10.1155/ije/7150482

**Published:** 2024-11-27

**Authors:** Ying Liu, Lei Gao, Min Li, Wei Zhang, Yan Wang, Jian Zhao

**Affiliations:** ^1^Department of Medical Imaging, Hebei Medical University Third Hospital, Qiaoxi District, Shijiazhuang 050051, Hebei, China; ^2^Department of Endocrinology, Hebei Medical University Third Hospital, Qiaoxi District, Shijiazhuang 050051, Hebei, China

## Abstract

**Aims:** To explore the distribution of site-specific volumetric bone mineral density (vBMD) and analyze the mechanism of vertebral compression fractures with type 2 diabetes mellitus (T2DM) subjects using quantitative computed tomography (QCT).

**Materials and Methods:** 304 postmenopausal women without T2DM and 274 postmenopausal women with T2DM underwent QCT scan, and all divided into three age subgroups. L1 vertebra was segmented into nine zones based on the corresponding position to the human body.

**Results:** Whether in the T2DM or non-T2DM of each age group, from the ventral to the dorsal side of L1 vertebra, the posterior third zones were the highest, and from the head to the foot of L1 vertebra, the middle third zones were the highest (*p* < 0.05). Global and most zonal vBMDs of T2DM were higher than those of non-T2DM in the age group of 50–59 years old, vBMD-mp of T2DM was higher in the age group of 60-59 years old, and vBMD-mm of T2DM was higher in the age group of 70–80 years old (*p* < 0.05). Zonal vBMDs in T2DM were higher than non-T2DM and the difference decreases with age especially in the upper third of L1 vertebra and the lower third of L1 vertebra.

**Conclusions:** Vertebral compression fractures and the confusion between T2DM and vBMD may be all caused by the heterogeneous distribution of vBMDs. The higher risk of T2DM with vertebral compression fractures may be associated with the different loss rate of global and site-specific vBMD, independent of vBMD itself.

## 1. Introduction

Diabetes and osteoporosis are common chronic disorders with increasing incidence among the elderly. A recent study indicated that osteoporosis is a serious complication of diabetes [1]. Osteoporosis is a disease characterized by the loss of bone density and deterioration of bone microstructure, resulting in reduced bone strength and increased risk of fractures. Osteoporotic fractures are very common, and usually caused by low-energy trauma, affecting every second woman and every fourth man over the age of 50 [2], and thoracolumbar region is a common site for compression fractures in osteoporotic vertebrae. Quantitative computed tomography (QCT) can measure the trabecular volumetric bone mineral density (vBMD), confirming the diagnosis of osteoporosis and predicting future fracture risk, avoiding the influence of vertebral osteophyte, facet degeneration, intervertebral disc stenosis, endplate sclerosis, and abdominal aortic wall calcification. Vertebral compression fractures (VCFs) are the most prevalent type of osteoporotic fracture, and most VCFs are clinically occult. In fact, approximately one-third of vertebral fragility fractures are clinically diagnosed, and only small percentage require hospitalization. Back pain is the main symptom, occurring in 85% of patients [3].

Type 2 diabetes mellitus (T2DM) is characterized by chronic hyperglycemia and insulin resistance, which affects bone metabolism. Available data suggested a higher risk of VCFs, particularly morphological vertebral fractures, in one third of postmenopausal women with T2DM [4–6]. However, the relationship between T2DM and vBMD remained controversial across studies compared with non-T2DM. Lin et al. showed that vertebral vBMD was lower in T2DM than non-T2DM [7]. Wang et al. showed that hyperglycemia is not associated with higher vBMD [8]. Gao *L* et al. showed that vertebral vBMD was higher in T2DM than non-T2DM [9]. de Waard et al. showed that T2DM has a normal to increased vBMD [10]. Although the vBMD of T2DM is partially contradictory and controversial, it is consistent that the fracture risk of T2DM is higher than those without T2DM. A meta-analysis of 15 published studies and cohorts showed that individuals with T2DM are at a higher risk of developing VCFs compared to those without T2DM [11]. Increased, decreased, or nondifferential vBMD and higher risk of fracture in T2DM patients is confusing. In general, we can understand that patients with low BMD or osteoporosis have an increased risk of fractures, as stated in a meta-analysis, which showed that osteoporosis was the risk factor of VCFs [12]. However, why is the fracture risk also high in T2DM when their vBMD is equal, or even higher than that of non-T2DM?

The relationship between vertebral vBMD variation and higher risk of VCFs remains to be further explored. QCT can measured the site-specific vBMD, and studies have shown that site-specific BMD measurements had more predictive value for complications after spinal surgery than standard whole-region measurements [13, 14]. These inconsistent results of BMD in T2DM compared to non-T2DM might be owing to limitations such as small study samples, different measurement location of BMD, and other confounding factors. Nevertheless, previous studies indicated significant differences in BMD according to vertebral level and among anatomical regions within each vertebra [15, 16]. To date, few data are available regarding the distribution of site-specific vBMD and higher risk of fracture in T2DM patients. The purpose of our study was to explore the distribution of site-specific vBMD in different vertebral regions of middle-aged and elderly women and analyze the mechanism of VCFs with T2DM using QCT.

## 2. Materials and Methods

### 2.1. Patient sample

**Figure 1:**
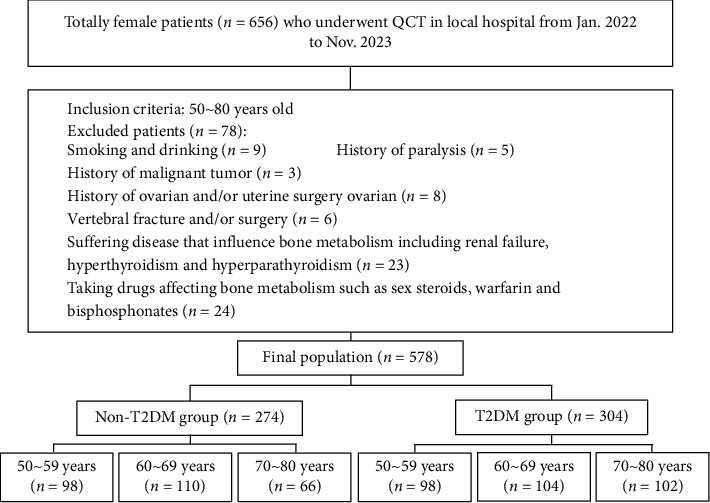
Flowchart illustrated the patient inclusion and selection process of the study. QCT = quantitative CT, non-T2DM = without type 2 diabetes mellitus, T2DM = type 2 diabetes mellitus.

We retrospectively reviewed the QCT examination from January 2022 to November 2023 with approval from the institutional review board (W2022-031-1). All participants provided written informed consent before QCT examination. Women, aged from 50 to 80 years old, were included with or without clinically diagnosed T2DM. Exclusion criteria included smoking and alcohol consumption, history of paralysis, malignancy, ovarian and/or uterine surgery, VCFs and/or surgery, and medical conditions and/or taking medications that affect bone metabolism. The T2DM patients receiving treatment of antihyperglycemic medications (e.g., metformin, glimepiride, pioglitazone, acarbose, and/or insulin). Finally, we included 578 women patients (64.0 ± 8.47 years, from 50 to 80 years), and 78 patients were excluded (Figure 1). All participants were divided into T2DM and non-T2DM groups, and then divided into three age subgroups at 10-year intervals (50–59 years old, 60–69 years old, and 70–80 years old). Age, height, and weight were recorded, and body mass index (BMI) was calculated as weight (kg) divided by height squared (m^2^).

### 2.2. Image Acquisition

All participants underwent QCT scan in the supine position by using CT scanner (Somatom Sensation 64, Siemens, Erlangen, Germany) with hands above the head and Mindways QCT phantom (Mindways Software Inc., Austin, TX, USA) closely dorsal side of each patient. CT scan was performed using the following parameters: tube voltage = 120 kV, tube current = 125 mAs, table height = 168 cm, matrix = 512 × 512, slice thickness = 1 mm, and field of view = 500 × 500 mm.

### 2.3. Image Analysis

**Figure 2:**
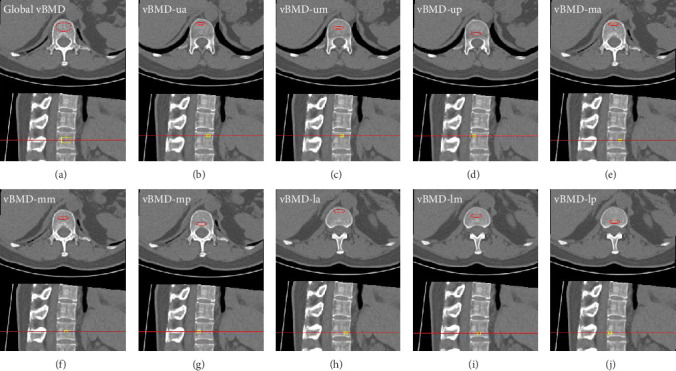
ROIs setting method of global and nine zonal vBMD. (a) Showed the semiautomatically ROI setting method of global vBMD at L1 by Mindways Software. ((b)–(j)): showed the semiautomatically and manually ROI setting methods of nine zonal vBMD at L1 by Mindways Software of a female women. Based on the sagittal and axial image, L1 is divided into nine zones, the upper and anterior (ua, vBMD abbreviated to vBMD-ua), upper and middle (um, vBMD abbreviated to vBMD-um), upper and posterior (up, vBMD abbreviated to vBMD-up), middle and anterior (ma, vBMD abbreviated to vBMD-ma), middle and middle (mm, vBMD abbreviated to vBMD-mm), middle and posterior (mp, vBMD abbreviated to vBMD-mp), lower and anterior (la, vBMD abbreviated to vBMD-la), lower and middle (lm, vBMD abbreviated to vBMD-lm), and lower and posterior (lp, vBMD abbreviated to vBMD-lp). ROI = region of interest, vBMD = volume bone mineral density, L1 = first lumbar vertebral body.

Images were transferred into the QCT workstation and analyzed using the Mindways software. Global vBMD (mg/cm^3^) was measured with an elliptical region of interest (ROI) about 250 mm^2^ area and 9 mm height placed at the midplane of L1 avoiding the cortical bone and hyperostosis osteosclerosis. L1 was divided into nine zones based on the corresponding position to the human body: the upper and anterior (ua, vBMD-ua), upper and middle (um, vBMD-um), upper and posterior (up, vBMD-up), middle and anterior (ma, vBMD-ma), middle and middle (mm, vBMD-mm), middle and posterior (mp, vBMD-mp), lower and anterior (la, vBMD-la), lower and middle (lm, vBMD-lm), and lower and posterior (lp, vBMD-lp) third region of L1 vertebral body, with an elliptical ROI, about 80 mm^2^ area, and 3 mm height placed at each zone, respectively (Figure 2) [17]. The global and nine zonal vBMDs were assessed by one analyst (Y.L., with 7 years of experience in QCT research).

### 2.4. Statistical Analysis

Statistical analyses were performed using SPSS version 26.0. and RStudio Mozilla/5.0. Values are means ± SD unless otherwise indicated. Pearson correlation test was used to test the correlation between vBMDs and age, and between vBMDs and BMI. Paired samples *t*-test was used to compare the pairwise differences among nine zonal vBMDs. Independent samples *t*-test was used to compare the difference of global vBMD between T2DM group and non-T2DM group, and independent samples *t*-test was used to compare the differences of nine zonal vBMDs between non-T2DM group and T2DM group. *p* < 0.05 was considered significant.

## 3. Results

### 3.1. Patients' Characteristics, Global vBMD, and Nine Zonal vBMDs Distribution

Age, BMI, global vBMD, and nine zonal vBMD distributions in non-T2DM group and T2DM group of three age subgroups were listed (Table 1). Global vBMD and nine zonal vBMD were moderate negatively correlated with age in non-T2DM group and T2DM group, respectively (Table 2). vBMD-ua and vBMD-la were negatively correlated with BMI in non-T2DM group (Table 2).

### 3.2. Pairwise Comparison Among Nine Zonal vBMDs

**Figure 3:**
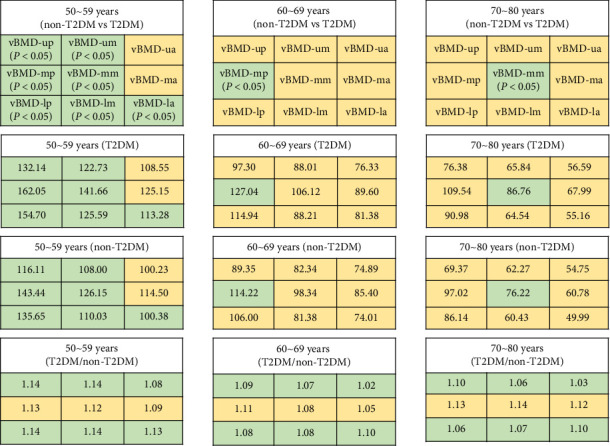
The 9 compartments in the first row represented the nine zones of the L1 vertebra, and the grid area with a green background indicated that there was statistically significant difference of corresponding zonal vBMD between non-T2DM and T2DM. The 9 compartments in the second row represented the mean values of each zonal vBMD corresponding to the first row in T2DM. The 9 compartments in the third row represented the mean values of each zonal vBMD corresponding to the first row in non-T2DM. The 9 compartments in the fourth row represented each zonal mean vBMD ratio of T2DM to non-T2DM corresponding to the first row. *P*: independent samples *t*-test between non-T2DM group and T2DM group. vBMD = volume bone mineral density, non-T2DM = without type 2 diabetes mellitus, T2DM = type 2 diabetes mellitus.

From the ventral to the dorsal side of L1 vertebra, vBMD-ua and vBMD-um were all lower than vBMD-up, vBMD-ma and vBMD-mm were all lower than vBMD-mp, and vBMD-la and vBMD-lm were all lower than vBMD-lp, in each age group whether in the T2DM group or non-T2DM group, respectively (Table 1 and Figure 3).

From the head to the foot of L1 vertebra, vBMD-ua and vBMD-la were all lower than vBMD-ma, vBMD-um and vBMD-lm were all lower than vBMD-mm, and vBMD-up and vBMD-lp were all lower than vBMD-mp, in each age subgroup whether in the T2DM group or non-T2DM group, respectively (Table 1 and Figure 3).

### 3.3. Comparison of vBMDs Between the T2DM Group and the Non-T2DM Group

In the group of 50–59 years old, there were differences of global vBMD, vBMD-um, vBMD-up, vBMD-mm, vBMD-mp, vBMD-la, vBMD-lm, and vBMD-lp between non-T2DM group and T2DM group (Table 1 and Figure 3). In the group of 60–69 years old, there was difference of vBMD-mp between non-T2DM group and T2DM group (Table 1 and Figure 3). In the group of 70–80 years old, there was difference of vBMD-mm between non-T2DM group and T2DM group (Table 1 and Figure 3).

### 3.4. Relationship Between Age and vBMDs Difference in T2DM Versus Non-T2DM

**Figure 4:**
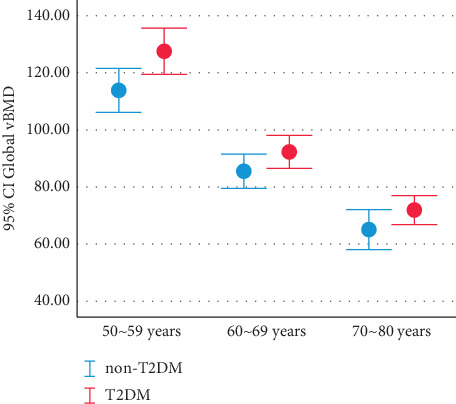
Error bar showed the distribution of global vBMD categorized by age group in non-T2DM and T2DM. vBMD = volume bone mineral density, non-T2DM = without type 2 diabetes mellitus, T2DM = type 2 diabetes mellitus.

**Figure 5:**
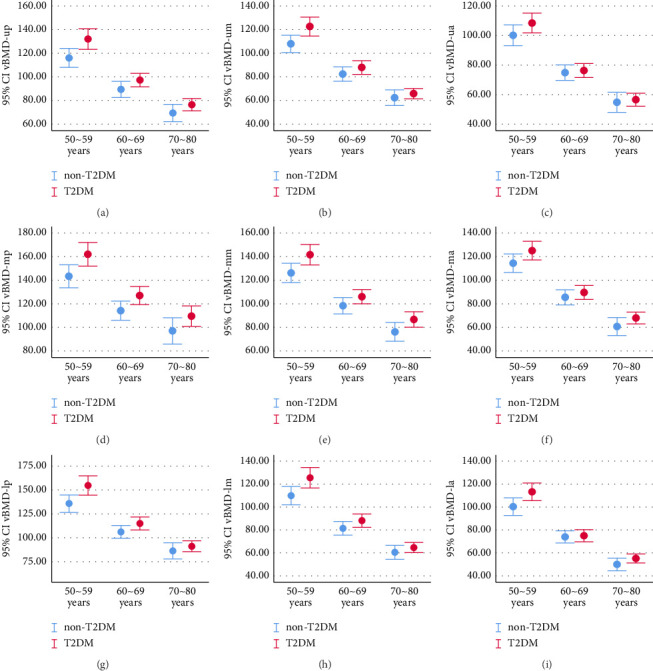
Error bar showed the distribution of zonal vBMD categorized by age group in non-T2DM and T2DM, which interpreted that zonal vBMD in T2DM were higher than non-T2DM and the difference decreases with age especially in the upper third of L1 vertebra (vBMD-ua, vBMD-um, and vBMD-up) and the lower third of L1 vertebra (vBMD-la, vBMD-lm, and vBMD-lp). vBMD = volume bone mineral density, non-T2DM = without type 2 diabetes mellitus, T2DM = type 2 diabetes mellitus.

The global vBMDs of each age group in T2DM were higher than non-T2DM and the difference decreased with age (Figure 4). Zonal vBMDs with T2DM were higher than those without T2DM and the difference decreases with age especially in the upper third of L1 vertebra (vBMD-ua, vBMD-um, and vBMD-up) and the lower third of L1 vertebra (vBMD-la, vBMD-lm, and vBMD-lp) (Figures 3 and 5).

## 4. Discussion

Low-energy traumatic osteoporotic VCFs is a typical manifestation of osteoporosis [18], resulting back pain, substantial VCFs (including three types: crush, wedge, and biconcave) and reduced the quality of life [19, 20], and with the aging, the prevalence of VCFs has been increasing annually [21]. The present study showed that the distribution of zonal vBMDs of L1 vertebral body was heterogeneous. Whether in the T2DM group or non-T2DM group of each age group, the posterior third zones were the highest compared with the anterior and middle third zones (vBMD-ua and vBMD-um compared with vBMD-up, vBMD-ma, and vBMD-mm compared with vBMD-mp, and vBMD-la and vBMD-lm compared with vBMD-lp) from the ventral to the dorsal side of L1 vertebra, which may quantitatively explain the vertebral wedge deformity from the perspective of vBMD. The middle third zones were the highest compared with the upper and lower third zones (vBMD-ua and vBMD-la compared with vBMD-ma, vBMD-um and vBMD-lm compared with vBMD-mm, and vBMD-up and vBMD-lp compared with vBMD-mp) from the head to the foot of L1 vertebra, which may quantitatively explain the vertebral biconcave deformity from the perspective of vBMD. The wedge and/or biconcave VCFs may be caused by the heterogeneous distribution of zonal vBMDs.

In our study, compared with non-T2DM patients, T2DM patients aged 50–59 years old had significantly higher global vBMD than non-T2DM patients, while there was no statistically significant difference in global vBMD between T2DM patients aged 60–69 years old and 70–80 years old. These results indicated that there was age-specific difference in global vBMD between T2DM and non-T2DM. There are differences of vertebral zonal vBMDs distribution between T2DM and non-T2DM. Most zonal vBMDs (vBMD-um, vBMD-up, vBMD-mm, vBMD-mp, vBMD-la, vBMD-lm, and vBMD-lp) of the T2DM were higher than those of the non-T2DM in the group of 50–59 years old, while only vBMD-mp of the T2DM group was higher in the group of 60–69 years old, and only vBMD-mm of the T2DM group was higher in the group of 70–80 years old, which showed that the numbers of zones existing vBMD difference between T2DM and non-T2DM were gradually decreased with age. From Figure 3, we can see that the difference in zonal vBMDs between T2DM and non-T2DM were gradually decreased. Zonal numbers with statistic vBMD difference and the range of zonal vBMDs difference were both gradually decreased with age when the T2DM group compared with the non-T2DM group. By now, the relationship between T2DM and vBMD is complex and has not yet been fully elucidated. These inconsistent findings may be related to vast differences in study design, vBMD measurement technology, difference in site of vBMD examination, selection of patients, and presence or absence of complications.

The vBMD loss in women consists of two stages, namely, a slow and persistent age-related loss and a rapid oestrogen-dependent process after menopause [2, 22]. Compared with non-T2DM, the vBMD loss was affected by DM [23]. In present study, the global mean vBMD ratio of T2DM to non-T2DM was 1.12 in the age group of 50–59; in the age group of 60–69, the ratio was 1.08; and in the age group of 70–80, the ratio was 1.10. The average global vBMD ratios of T2DM to non-T2DM in the age group of 60–69 and 70–80 were lower than 50–59. If the same rate of bone loss occurs, the vBMD ratio of T2DM to non-T2DM should remain the same, while the values were decreasing, which interpreted that the global vBMD of T2DM group is losing faster than that of non-T2DM group. Zonal vBMD in T2DM were higher than non-T2DM, and each zonal mean vBMD ratio of T2DM to non-T2DM were all decreased in the group of 60–69 years old and the group of 70–80 years old compared to the group of 50–59 years old, especially in the upper third of L1 vertebra (vBMD-ua, vBMD-um, and vBMD-up, Figures 3 and 5(a), 5(b), and 5(c)) and the lower third of L1 vertebra (vBMD-la, vBMD-lm, and vBMD-lp, Figures 3 and 5(g), 5(h), and 5(i)). Increased risk of VCFs in T2DM patients, whether may be explained by the increased vBMD loss at the upper and lower edges of the vertebral body, independent of vBMD itself.

Our study has several limitations. Firstly, we only adjusted for age and BMI, and other factors known to affect bone health (physical activity, vitamin D levels, HbA1c levels, and antihyperglycemic medication) and the duration of T2DM, which could potentially confound the relationship between T2DM and vBMD, were not included in the analysis, and we will further investigate in the future. Secondly, we analyzed nine zonal vBMD in only L1 semiautomatically. However, with the development of computer AI-assisted technology, it may be possible to make more zones of the vertebrae and multiple vertebrae in the future. Finally, there was no data on vertebral fractures, and VCFs is a biomechanical process, we had only studied the vBMD, and other factors (like bone microstructure, trabecular thickness, trabecular number, bone strength, and bone metabolism) have not been taken into consideration.

In conclusion, VCFs (wedge and/or biconcave) may be all caused by the heterogeneous distribution of vBMDs in vertebral body, and the confusion between T2DM and vBMD may be due to the heterogeneous distribution of vBMDs in vertebral body. The higher risk of T2DM with VCFs may be associated with the different loss rate of global and site-specific vBMD, independent of vBMD itself.

## Figures and Tables

**Table 1:** Patients' characteristics and vBMD distribution in non-T2DM group and T2DM group of three age subgroups.

Parameter	All patients	Non-T2DM group	T2DM group	*t/P * ^∗^
50–59 years (*n*)	196	98	98	
Age (y)	54.63 ± 3.07	54.61 ± 3.05	54.64 ± 3.10	−0.070/0.945
BMI (kg/m^2^)	24.60 ± 3.83	24.08 ± 3.48	25.12 ± 4.09	−1.909/0.058
Global vBMD (mg/cm^3^)	120.72 ± 39.86	113.87 ± 38.25	127.56 ± 40.45	**−2.434/0.016**
vBMD-ua (mg/cm^3^)	104.39 ± 34.63	100.23 ± 35.35^#,₸^	108.55 ± 33.57^#,₸^	−1.690/0.093
vBMD-um (mg/cm^3^)	115.37 ± 39.14	108.00 ± 36.69^#,₶^	122.73 ± 40.30^#,₶^	**−2.674/0.008**
vBMD-up (mg/cm^3^)	124.12 ± 42.55	116.11 ± 40.24^₳^	132.14 ± 43.47^₳^	**−2.679/0.008**
vBMD-ma (mg/cm^3^)	119.83 ± 39.38	114.50 ± 39.12^$^	125.15 ± 39.11^$^	−1.906/0.058
vBMD-mm (mg/cm^3^)	133.90 ± 42.73	126.15 ± 40.85^$^	141.66 ± 43.37^$^	**−2.576/0.011**
vBMD-mp (mg/cm^3^)	152.75 ± 49.77	143.44 ± 48.48	162.05 ± 49.53	**−2.658/0.009**
vBMD-la (mg/cm^3^)	106.83 ± 38.51	100.38 ± 38.42^&,₸^	113.28 ± 37.70^&,₸^	**−2.374/0.019**
vBMD-lm (mg/cm^3^)	117.81 ± 42.58	110.03 ± 39.48^&,₶^	125.59 ± 44.31^&,₶^	**−2.595/0.010**
vBMD-lp (mg/cm^3^)	145.17 ± 48.92	135.65 ± 45.54^₳^	154.70 ± 50.53^₳^	**−2.771/0.006**
60∼69 years (*n*)	214	110	104	
Age (y)	64.13 ± 2.83	64.13 ± 2.80	64.13 ± 2.89	−0.019/0.985
BMI (kg/m^2^)	25.05 ± 3.54	24.66 ± 3.48	25.45 ± 3.58	−1.642/0.102
Global vBMD (mg/cm^3^)	88.82 ± 30.85	85.53 ± 31.80	92.31 ± 29.57	−1.613/0.108
vBMD-ua (mg/cm^3^)	75.59 ± 26.23	74.89 ± 27.79^#,₸^	76.33 ± 24.59^#,₸^	−0.401/0.689
vBMD-um (mg/cm^3^)	85.10 ± 30.82	82.34 ± 32.02^#,₶^	88.01 ± 29.37^#,₶^	−1.347/0.179
vBMD-up (mg/cm^3^)	93.21 ± 33.08	89.35 ± 36.08^₳^	97.30 ± 29.21^₳^	−1.765/0.079
vBMD-ma (mg/cm^3^)	87.44 ± 32.54	85.40 ± 34.47^$^	89.60 ± 30.39^$^	−0.942/0.347
vBMD-mm (mg/cm^3^)	102.12 ± 34.08	98.34 ± 36.59^$^	106.12 ± 30.88^$^	−1.678/0.095
vBMD-mp (mg/cm^3^)	120.45 ± 41.66	114.22 ± 42.68	127.04 ± 39.71	**−2.272/0.024**
vBMD-la (mg/cm^3^)	74.52 ± 27.30	74.01 ± 28.19^&,₸^	81.38 ± 31.22^&,₸^	−0.281/0.779
vBMD-lm (mg/cm^3^)	84.70 ± 30.52	81.38 ± 31.22^&,₶^	88.21 ± 29.51^&,₶^	−1.642/0.102
vBMD-lp (mg/cm^3^)	110.35 ± 35.21	106.00 ± 34.78^₳^	114.94 ± 35.24^₳^	−1.868/0.063
70∼80 years (*n*)	168	66	102	
Age (y)	74.67 ± 3.02	74.59 ± 3.00	74.72 ± 3.05	−0.260/0.795
BMI (kg/m^2^)	24.55 ± 3.44	24.28 ± 3.64	24.72 ± 3.30	−0.803/0.423
Global vBMD (mg/cm^3^)	69.25 ± 27.02	65.09 ± 28.45	71.94 ± 25.84	−1.614/0.109
vBMD-ua (mg/cm^3^)	55.87 ± 24.39	54.75 ± 27.53^#,₸^	56.59 ± 22.24^#,₸^	−0.477/0.634
vBMD-um (mg/cm^3^)	64.44 ± 24.51	62.27 ± 26.96^#,₶^	65.84 ± 22.82^#,₶^	−0.921/0.359
vBMD-up (mg/cm^3^)	73.63 ± 28.11	69.37 ± 30.07^₳^	76.38 ± 26.56^₳^	−1.585/0.115
vBMD-ma (mg/cm^3^)	65.16 ± 27.69	60.78 ± 30.37^$^	67.99 ± 25.56^$^	−1.658/0.099
vBMD-mm (mg/cm^3^)	82.62 ± 32.66	76.22 ± 32.05^$^	86.76 ± 32.53^$^	**−2.064/0.041**
vBMD-mp (mg/cm^3^)	104.62 ± 44.51	97.02 ± 45.15	109.54 ± 43.60	−1.792/0.075
vBMD-la (mg/cm^3^)	53.13 ± 21.08	49.99 ± 22.49^&,₸^	55.16 ± 19.96^&,₸^	−1.560/0.121
vBMD-lm (mg/cm^3^)	62.93 ± 23.23	60.43 ± 24.53^&,₶^	64.54 ± 22.32^&,₶^	−1.122/0.263
vBMD-lp (mg/cm^3^)	89.08 ± 30.51	86.14 ± 33.60^₳^	90.98 ± 28.35^₳^	−1.004/0.317

*Note:* vBMD-ua, vBMD-um, vBMD-up, vBMD-ma, vBMD-mm, vBMD-mp, vBMD-la, vBMD-lm, and vBMD-lp are the abbreviations of vBMDs for the upper and anterior, upper and middle, upper and posterior, middle and anterior, middle and middle, middle and posterior, lower and anterior, lower and middle, and lower and posterior third zones of L1. The bold values presented are statistically different between the non-T2DM group and T2DM group.

Abbreviations: Global vBMD, global volume bone mineral density of the first lumbar vertebral body (L1); T2DM, type 2 diabetes mellitus; vBMD, volume bone mineral density.

^∗^Independent samples *t*-test between non-T2DM group and T2DM group.

^#^vBMD-ua and vBMD-um compared with vBMD-up, respectively, and there were differences.

^$^vBMD-ma and vBMD-mm compared with vBMD-mp, respectively, and there were differences.

^&^vBMD-la and vBMD-lm compared with vBMD-lp, respectively, and there were differences.

^₸^vBMD-ua and vBMD-la compared with vBMD-ma, respectively, and there were differences.

^₶^vBMD-um and vBMD-lm compared with vBMD-mm, respectively, and there were differences.

^₳^vBMD-up and vBMD-lp compared with vBMD-mp, respectively, and there were differences.

**Table 2:** Correlation between vBMD and age and BMI in non-T2DM group and T2DM group.

		Global vBMD	vBMD-ua	vBMD-um	vBMD-up	vBMD-ma	vBMD-mm	vBMD-mp	vBMD-la	vBMD-lm	vBMD-lp
Non-T2DM	Age (*r* [*P*])	**−0.538 (< 0.001)**	**−0.547 (< 0.001)**	**−0.538 (< 0.001)**	**−0.510 (< 0.001)**	**−0.553 (< 0.001)**	**−0.506 (< 0.001)**	**−0.409 (< 0.001)**	**−0.577 (< 0.001)**	**−0.566 (< 0.001)**	**−0.514 (< 0.001)**
	BMI (*r* [*P*])	−0.084 (0.163)	**−0.136 (0.024)**	−0.083 (0.169)	−0.067 (0.269)	−0.108 (0.076)	−0.066 (0.277)	−0.031 (0.605)	**−0.133 (0.028)**	−0.115 (0.058)	−0.082 (0.176)

T2DM	Age (*r* [*P*])	**−0.567 (< 0.001)**	**−0.621 (< 0.001)**	**−0.590 (< 0.001)**	**−0.554 (< 0.001)**	**−0.596 (< 0.001)**	**−0.521 (< 0.001)**	**−0.419 (< 0.001)**	**−0.648 (< 0.001)**	**−0.604 (< 0.001)**	**−0.561 (< 0.001)**
	BMI (*r* [*P*])	0.030 (0.601)	0.032 (0.579)	0.055 (0.343)	0.064 (0.265)	0.028 (0.623)	0.051 (0.379)	0.058 (0.313)	0.017 (0.768)	0.036 (0.534)	0.040 (0.491)

*Note:* vBMD-ua, vBMD-um, vBMD-up, vBMD-ma, vBMD-mm, vBMD-mp, vBMD-la, vBMD-lm, and vBMD-lp are the abbreviations of vBMD for the upper and anterior, upper and middle, upper and posterior, middle and anterior, middle and middle, middle and posterior, lower and anterior, lower and middle, and lower and posterior third zones of L1. *r:* the Spearman correlation coefficient. The bold values presented are statistically different in Spearman correlation analysis.

Abbreviations: BMI, body mass index; Global vBMD, global volume bone mineral density of first lumbar vertebral body (L1); Non-T2DM, without type 2 diabetes mellitus; T2DM, type 2 diabetes mellitus; vBMD, volume bone mineral density.

## Data Availability

Data generated and/or analyzed during the current study are not publicly available. Researchers with a specific question regarding the study are encouraged to contact the corresponding authors.
